# *Bacilli* the most important biocontrol agent in Brazilian agriculture: from mechanisms of action to market adoption

**DOI:** 10.1007/s42770-026-02096-3

**Published:** 2026-09-28

**Authors:** Wagner Bettiol, Roberto Lanna-Filho, Cristiano Limberger

**Affiliations:** 1 Embrapa Meio Ambiente, Rod. SP-340 Km 127,5, Jaguariúna, SP 13.918-110 Brazil; 2 Departamento de Fitossanidade, Universidade Federal do Rio Grande do Sul, Porto Alegre, RS 91540-000 Brazil; 3 Kynothec Brasil, R. Dr. Eraldo Aurélio Franzese, 184, Valinhos, SP 13271-608 Brazil

**Keywords:** Biological control, Brazilian biocontrol market, *Bacillus*-based products

## Abstract

*Bacilli* have become one of the most commercially significant groups of microorganisms used in agricultural biocontrol products in Brazil. Their ability to form endospores and produce a wide range of metabolites allows them to control plant diseases, promote plant growth, and alleviate stress, supporting their extensive adoption in crop protection systems. This study analyzes the Brazilian market for *Bacillus*-based products used in plant disease management and summarizes their mechanisms of action. Data on commercial registrations, active strains, product formulations, and target diseases were compiled from Brazilian national regulatory databases. Market indicators, such as potential area treated (PAT) and turnover, were estimated using structured agricultural surveys conducted across Brazil during the 2019/2020 and 2024/2025 crop seasons, the latter encompassing more than 16,000 interviews with farmers and agricultural managers. By December/2025, 297 *Bacillus*-based products containing 126 strains were commercialized in Brazil, with *Bacillus amyloliquefaciens*, *B. subtilis*, *B. velezensis*, and *B. pumilus* being the most prominent species. These products are applied via seed treatment, soil drenching, furrow, and foliar spraying to manage fungal, bacterial, and nematode diseases. During the analyzed period, PAT increased from 7.5 to 37.2 million hectares (391% growth) and turnover increased from US$114.31 to US$403.17 million (252% growth). Currently, *Bacillus*-based products accounted for approximately 48% of the Brazilian biological control market, highlighting the prevalence of this genus in the sector. Overall, these results demonstrate how advances in microbial biotechnology, regulatory development, and farmer adoption have positioned *Bacillus* spp. as key components of the rapidly expanding biocontrol industry.

## Introduction

*Bacilli* are renowned for their versatility in suppressing a wide range of plant pathogens, promoting plant growth, and alleviating abiotic stresses [1–19]. This multifunctionality is closely associated with their metabolic diversity and ecological adaptability.

*Bacillus* spp. produce endospores, which are extremely resistant structures able to withstand several adverse environmental conditions [20, 21]. This sporulation capacity enables these bacteria to survive, persist, and remain metabolically viable under unfavorable conditions, allowing these bacteria to thrive across a wide range of environments and enhancing their effectiveness as biocontrol and plant growth-promoting agents [22, 23].

As bioactive constituents of commercial formulations, endospore-based products offer enhanced flexibility of use by preserving bacterial cell viability under exposure to a broad range of chemical agents, thermal extremes, and water deficit conditions [24–28]. From a practical standpoint, endospore-based products exhibit wide compatibility with different synthetic fungicides, herbicides, and insecticides [29–33], which enables their co-application through tank mixing prior to field application. Moreover, *Bacillus* species are able to survive in crop debris by synthesizing enzymatic complexes capable of degrading plant straw and acting against pathogens [11, 34–36]. This ability makes *Bacillus* species highly competitive and well suited to establish in specific niches within intensive agricultural systems, where these bacteria can exert multiple mechanisms of action against pathogens [37, 38].

The mechanisms of action employed by *Bacillus* species in the control of plant pathogens include antibiosis, colonization and competition for ecological niches, quorum-quenching, and induced resistance [39–43]. In terms of plant growth promotion, *Bacillus* acts through both direct or indirect mechanisms. Direct mechanisms include the production of phytohormones (indole-3-acetic acid, cytokinin, gibberellic acid), solubilize phosphorus and other nutrients, and the production of siderophores, which stimulate plant growth [44–48]. Indirect mechanisms include changes in root system architecture, which influence the plant’s ability to exploit the soil resources by improving water and nutrient uptake [49, 50].

Several species of *Bacillus* have been shown to improve plant tolerance to abiotic stress, such as salinity stress [51–54], drought stress [7], and thermal stress [55, 56]. Medeiros and Bettiol [12] reported increased levels of peroxidase, polyphenol oxidase, phenylalanine ammonia lyase, and L-proline in tomato plants treated with *Bacillus* grown under saline stress when compared to those grown without salt stress. Fonseca et al. [7] reported that sugarcane under drought stress inoculated with *Bacillus subtilis* improved plant nutrition and chlorophyll concentrations. Also, these authors reported that the gas exchange parameters, root growth, tillering, stalk weight, and higher sucrose concentration in the stalks were increased.

In Brazil, *Bacillus*-based formulations for biological control purposes account for approximately 80% of the number of products available on the market [57, 58]. Products for nematode control stand out, representing nearly 70% [57]. Among the 729 microbiological-based products registered for the control of fungi, bacteria, nematodes, and insects, the species *B. amyloliquefaciens* (7%), *B. thuringiensis* (6.2%), *B. subtilis* (4%), *B. velezensis* (2.2%), and *B. licheniformis* (1%) stand out as the principal active agents comprising commercial formulations [14, 57]. These findings reflect the global market scenario [59–61], which highlights the widespread adoption of formulations based on *Bacillus* strains for the biological control of pests and diseases across diverse crops of major economic importance worldwide [62, 63].

As a result of these well-documented physiological and biochemical benefits, *Bacillus* species have been widely commercialized for agriculture use, with numerous *Bacillus-*based products currently available worldwide. Given Brazil’s importance in the production and consumption of biocontrol agents [64–67], this review discusses the growth of the Brazilian market for *Bacillus*-based products in terms of both potential treated area and turnover. We also present data on the potential treated area and turnover of all commercial *Bacillus-*based products registered in Brazil between 2020 and 2025, in addition to outlining the mechanisms of action of *Bacillus* species in the control of plant diseases.

### Mechanisms of action of *Bacillus* to control plant disease

Widely used as biocontrol agents, *Bacillus* species exhibit a broad range of mechanisms that act either directly or indirectly against plant pathogens [68–71]. In addition to conventional biocontrol strategies - such as antibiosis, competition, and the induction of systemic resistance - *Bacillus* species can also activate the quorum quenching system. This process inhibits pathogenesis by interrupting the mechanisms of pathogen attack [72–75].

The antagonistic activity exerted by *Bacillus* species occurs dynamically and simultaneously on different pathogens structures, resulting in a multi-site mode capable of repressing or suppressing the pathogenic activity. Understanding these mechanisms and their role in pathogen suppression is fundamental for optimizing the application of *Bacillus-*based biocontrol agents and maximizing their effectiveness against major diseases affecting economically important crops. The primary mechanisms of action include antibiosis, colonization and competition for ecological niches, quorum quenching, and induced resistance.

- Antibiosis

The biocontrol activity of *Bacillus* species is largely attributed to their ability to produce a wide range of chemically diverse compounds that act directly against pathogens. *Bacilli* genomes are particularly rich in biosynthetic gene clusters encoding bioactive secondary metabolites, such as toxins, bacteriocins, (lipo)peptides, polyketides, volatile organic compounds (VOCs), and siderophores [76, 77]. Furthermore, compounds that may be associated with primary metabolism, such as (hydro)lytic enzymes, also exhibit bioactive action against pathogens [78–81].

Up to 12% of the *Bacillus* genome is dedicated to the biosynthesis of metabolites [82–84], contributing to their survival and ecological fitness. In *Bacillus subtilis* strains, approximately 4 to 5% of their genome is dedicated to metabolite biosynthesis [85], whereas *Bacillus amyloliquefaciens* strains may allocate about 8.5% [76, 77, 86]. For *Bacillus velezensis* strains, between 2.29 and 10% of the genome is devoted to the biosynthesis of antimicrobials [87, 88].

The primary antimicrobials produced by different *Bacillus* species are antibiotics [89], which are (lipo)peptides of either ribosomal or non-ribosomal nature [69]. As ribosomal peptides, bacteriocins exhibit a broad antibacterial activity by inducing cell lysis, forming pores in the plasma membrane, or inhibiting cell wall biosynthesis [90–92].

Non-ribosomal (lipo)peptides are an extremely heterogeneous group of compounds which can be linear or cyclic in nature. Among *Bacillus* spp., the most studied compounds are the cyclic lipopeptides (CLPs), which exhibit amphiphilic nature [93, 94]. The primary mechanisms of action of cyclic lipopeptides involve interactions with the pathogen’s plasma membrane, leading to structural changes and increased permeability through rupture, solubilization, or the formation of ion-conducting pores. The most important CLPs are represented by the iturins, fengycins, and surfactins families [85, 95, 96]. Iturins display potent antifungal activity but are less active against bacteria [69]. The main mode of action of these compounds is the formation of ion-conducting transmembrane channels, which promote electrolyte leakage and disruption of the pathogen’s plasma membrane.

The antagonistic activity of *Bacillus* species can also be associated with the synthesis of volatile organic compounds (VOCs). In bacteria, VOCs - which belong to different chemical classes - are generally produced via catabolic pathways, including glycolysis, proteolysis, and lipolysis [97, 98]. In *Bacillus*, VOCs typically contain up to 20 carbon atoms and belong to the following chemical classes: alcohols, aldehydes, acids, benzenoids, ketones, esters, hydrocarbons, pyrazines, sulfur compounds, and terpenes [99–102]. Volatile organic compounds synthesized by *Bacillus* species have demonstrated bioactivity against fungi, bacteria, and nematodes.

*Bacillus* species are capable of synthesizing a diverse range of hydrolytic enzymes with the potential to collapse the cell wall of fungi and oomycetes [103, 104] and to degrade the cuticle of nematodes [105–109]. This enzymatic complex is formed by chitinases, β-glucanases, chitosanases, proteases, lipases, and cellulases, which act to hydrolyze specific components of the cell wall by breaking the glycosidic bonds that link polymeric structures. As a result, the target organism loses key protective and functional properties, such as selective permeability, tensile strength, and turgor-driven cell expansion, all of which are essential for growth and host infection [110, 111].

Iron is an essential element for various biological processes such as oxygen metabolism, DNA and RNA synthesis, electron transfer, and enzymatic processes [112]. Although iron is the fourth most abundant element on Earth, its bioavailability in aerobic soils is quite limited [113, 114]. This is because the predominant form in nature, the ferric ion (Fe³⁺), has low solubility, hindering its assimilation by both bacteria and plants. As a result, the amount of iron available to living organisms is extremely low. To survive with such a limited iron supply, *Bacillus* species synthesize non-ribosomal metal-chelating peptides (siderophores) of low molecular weight (∼400–1500 Da) [114, 115]. These peptides display a high affinity for Fe³⁺ (with association constants ranging from of 10^23^ a 10^52^) and form Fe-siderophore complexes which are capable of binding to membrane receptors (FeuA) [116, 117]. Since bacterial siderophores have a higher affinity for Fe³⁺ than the uptake systems of many pathogens, they sequester the available iron in the environment. This results in iron deprivation for the pathogen, leading to the inhibition of mycelial growth, reduced sporulation, or even cell death [118, 119]. On the other hand, plant growth is generally not affected by this depletion of iron in the rhizosphere caused by siderophores because most plants can thrive at much lower iron concentrations than most microorganisms [120].

b. Colonization and ecological niche competition

**Fig. 1:**
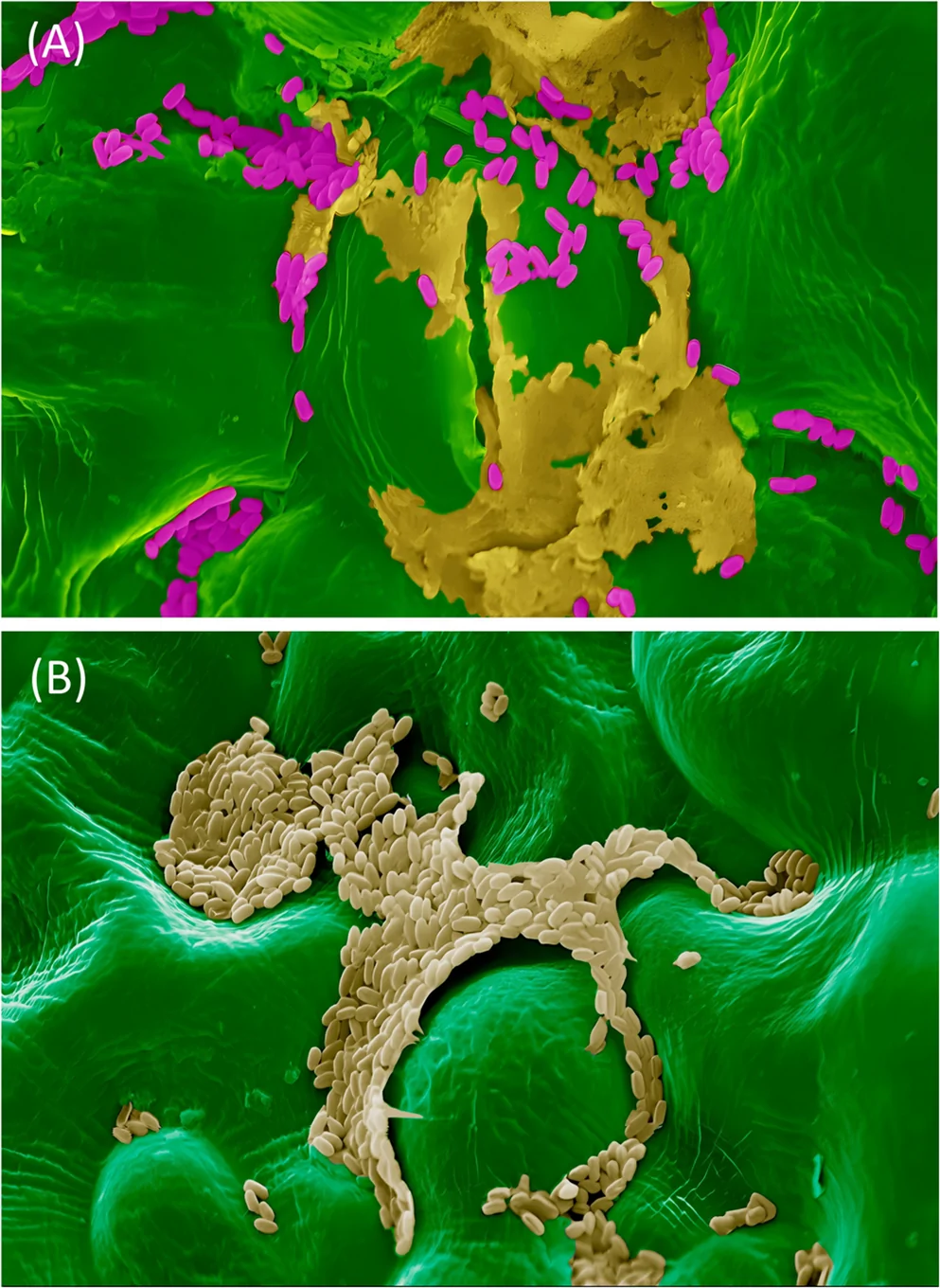
Bacterial cells aggregated on the surface of tomato leaves. (**A**) Aggregates of *Bacillus amyloliquefaciens* located adjacent to the stomatal region and over the stomatal pore (ostiole). The yellow-colored area corresponds to the stomatal exudate, whereas bacterial cells are shown in purple. (**B**) Aggregates of *Paenibacillus macerans* located at the junctions of the anticlinal walls of epidermal cells. Scanning electron micrographs were artificially colorized for illustrative purposes. Image courtesy of R. Lanna-Filho

The success of biocontrol provided by *Bacillus* species is based on their ability to colonize and to establish themselves in specific plant niches. The synthesis of compounds toxic to pathogens directly depends on a sufficiently high bacterial population density on the host. In cellular aggregates (Fig. 1), *Bacillus* stimulates the production of autoinducers which activate the expression of genes responsible for collective behaviors [121–123], including those responsible for the synthesis of bioactive molecules. In addition to creating an environment saturated with toxic compounds, a high bacterial population density also prevents pathogens from occupying the same space. *Bacillus* exhibits a faster growth rate compared to pathogens, providing a substantial competitive advantage in the occupation and colonization of specific niches. The radial growth (colonies) of *Bacilli* is around 2–5 mm after 24 h [124, 125], while pathogenic fungi grow on average 5–20 mm/day [126–130].

On the host surface, bacterial aggregates become embedded in a heterogeneous exopolymeric matrix (EPM), which protects the cells against adverse environmental conditions, such as toxic substances, desiccation, thermal extremes, pH changes, nutrient depletion, and solar irradiation [131–133]. Nutrients derived from the plant-microbe association accumulate within the biofilm, enabling the bacterial cells inside the exopolymeric matrix to remain viable for a long period. In addition, there is an accumulation of toxic compounds that permeate the aggregate, causing niche suppressiveness against phytopathogens. The occupation and colonization of spaces (anticlinal wall junctions, ostioles, substomatal chambers, and the base of trichomes) in the phylloplane prevent the establishment of pathogens and avert successful infection.

**Fig. 2:**
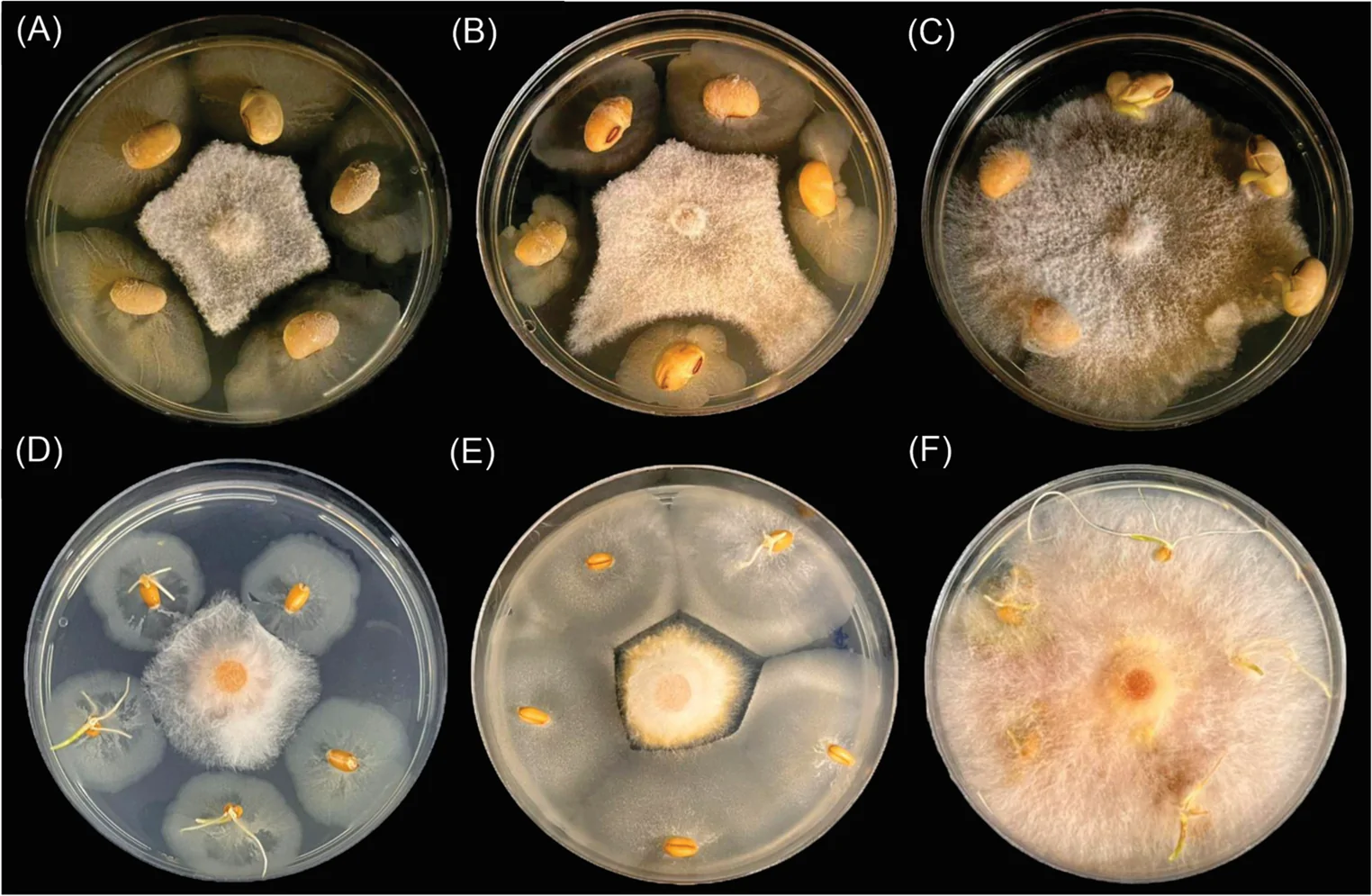
Soybean and wheat seeds treated with *Bacillus* spp. Inhibitory activity against *Diaporthe longicolla* promoted by the bacterial consortia: (**A**) *Bacillus amyloliquefaciens*, *Bacillus velezensis*, and *Bacillus thuringiensis*; (**B**) *Bacillus subtilis*, *B. velezensis*, and *Bacillus pumilus*. Corresponding inhibition of *Fusarium graminearum* is shown for (**D**) *B. amyloliquefaciens* and (**E**) *B. subtilis*, *B. velezensis*, and *B. pumilus*. Water-treated controls are shown in (**C**) and (**F**). Image courtesy of R. Lanna-Filho

In the rhizoplane, *Bacillus* species form extensive regions of cellular aggregates, with the most intense accumulation occurring near micro-wounds and root elongation zones (e.g., behind the root cap) [134–136]. By forming a robust biofilm layer on the roots, *Bacillus* establishes a physico-toxic barrier - a biofilm-based defense that combines physical exclusion of phytopathogens with localized toxic effects mediated by antimicrobial compounds - that restricts the penetration and colonization of root phytopathogens. Seeds treated with *Bacillus* spp. show a relevant protective effect against pathogens due to the rapid multiplication rate and bacterial colonization on the seed surface. In addition to the topical action, *Bacillus* promotes a suppressive effect that extends to the spermosphere (Fig. 2) and to the soil. At the moment of radicle protrusion, these bacteria form a dense biofilm in the rhizoplane, creating a persistent protective barrier against pathogens.

c. Quorum-quenching

An important biocontrol mechanism triggered by *Bacillus* species involves interference with the intraspecific communication of bacteria and fungi. This process, known as quorum-quenching, interrupts the signaling mediated by quorum-sensing, leading to disturbances in physiological processes such as metabolism, virulence, sporulation, and biofilm formation [137, 138]. The quorum-quenching phenomenon was first reported in *Bacillus thuringiensis*, through the synthesis of the AiiA enzyme, which inactivated the quorum-sensing signal of *Pectobacterium carotovorum* (syn.: *Erwinia carotovora*), thereby attenuating the pathogen’s virulence [139, 140]. Quorum-sensing is a cell-cell communication system that enables microorganisms to detect population density and collectively coordinate gene expression. In bacterial and fungal pathogens, quorum-sensing plays a central role in both pathogenicity and survival within the host [73, 141, 142]. Consequently, the destruction of quorum-sensing signaling molecules causes drastic effects on pathogenesis and compromises essential processes required for successful host colonization.

d. Induced resistance

The mechanism of induced resistance in plants can be triggered by cellular constituents or by compounds synthesized by *Bacillus* spp [143–146]. Plant defense responses, whether local or systemic, involve a complex network of interactions regulated by the pathways of salicylic acid (SA), jasmonic acid (JA), and ethylene (ET). The modulation of these pathways depends on proteins that exert synergistic, antagonistic, or cooperative effects among the mediators of Systemic Acquired Resistance (SAR) and Induced Systemic Resistance (ISR) [147–151]. The SAR pathway is SA-dependent, whereas the ISR pathway is JA/ET-dependent [152, 153]. Both defense mechanisms (SAR and ISR) can be activated by *Bacillus* spp., resulting in distal defense responses that provide plant protection in tissues spatially separated from the initial contact site of interaction [41, 154].

e. From mechanisms to integrated crop protection

The multiple mechanisms described above should not be viewed as independent biological phenomena, but rather as interconnected biological processes that collectively determine the agronomic performance of *Bacillus*-based products under field conditions. Unlike conventional synthetic pesticides, whose efficacy generally depends on one or a few specific biochemical targets, *Bacillus* spp. simultaneously combine direct pathogen suppression through antibiosis, ecological exclusion by rapid colonization and niche competition, disruption of pathogen communication via quorum-quenching, activation of induced systemic resistance, and enhancement of plant vigor through growth promotion and improved tolerance to abiotic stresses. Consequently, field performance results from the integration of ecological, physiological, biochemical, and plant-mediated interactions operating throughout the crop cycle rather than from a single antimicrobial activity [155–160].

This multifunctional mode of action positions *Bacillus*-based products as components of Integrated Crop Protection (ICP), where biological control agents are incorporated into comprehensive disease management programs together with resistant cultivars, crop rotation, cultural practices, decision-support systems, and, when necessary, synthetic pesticides [161, 162]. Within this framework, *Bacillus*-based products are primarily deployed as preventive components of disease management to reduce primary inoculum, delay epidemic development, suppress pathogen establishment, and enhance crop resilience before severe disease outbreaks occur. Their broad compatibility with numerous fungicides and other crop protection products further facilitates integration into existing production systems without requiring major changes in growers’ management practices [29–33].

A practical example is the association of *Bacillus*-based products with demethylation inhibitor (DMI) fungicides, such as triazoles [163]. During plant colonization, *Bacillus* rapidly establishes biofilms on the phylloplane and rhizoplane, creating a stable microbial barrier that favors persistence, niche occupation, and the localized accumulation of bioactive metabolites, including cyclic lipopeptides, hydrolytic enzymes, siderophores, and volatile organic compounds [121–133]. These compounds directly interfere with fungal cell integrity, particularly through membrane disruption and inhibition of pathogen development. In contrast, triazoles inhibit sterol 14α-demethylase (CYP51), blocking ergosterol biosynthesis and compromising fungal membrane formation. Thus, biological and chemical agents target different physiological processes of the pathogen, providing distinct yet convergent modes of action of action that enhance disease suppression while simultaneously diversifying the selection pressures imposed on pathogen populations, thereby contributing to fungicide resistance management [164–166].

Another important aspect of the integration between *Bacillus*-based products and synthetic fungicides concerns the interaction between *Bacillus*-mediated induced resistance and the direct activity of systemic fungicides [166, 167]. Whereas fungicides predominantly protect plant tissues through direct activity against the pathogen after translocation within the plant, *Bacillus*-mediated induced resistance activates endogenous defense pathways regulated by salicylic acid, jasmonic acid, and ethylene, resulting in systemic defense responses in tissues spatially separated from the initial colonization site [143–153]. Consequently, chemical fungicides act directly on the pathogen, whereas *Bacillus* simultaneously enhances the defensive capacity of the host, generating a complementary protection strategy that combines direct antimicrobial activity with increased plant immune competence. This functional integration represents one of the principal reasons why *Bacillus*-based products have become increasingly incorporated into integrated crop protection programs worldwide.

An additional advantage of *Bacillus*-based products lies in their multisite biological activity. Because pathogen suppression results from the simultaneous action of several complementary mechanisms rather than from a single molecular target, these products impose substantially lower selection pressure for resistance than site-specific fungicides. Consequently, their incorporation into integrated disease management programs contributes not only to disease suppression but also to fungicide resistance management by diversifying selection pressures within pathogen populations [166, 167]. Taken together, these characteristics demonstrate that the value of *Bacillus*-based products extends well beyond their antimicrobial activity. Their multifunctionality, compatibility with conventional crop protection technologies, low selection pressure for resistance, and ability to simultaneously suppress pathogens, stimulate plant growth, and enhance crop resilience position them as biological components of modern ICP programs rather than as stand-alone alternatives to synthetic pesticides. Consequently, the continued expansion of *Bacillus*-based products is expected to be driven not only by advances in microbial biotechnology [159, 168], together with the increasing adoption of integrated and sustainable disease-management strategies aimed at maintaining crop productivity, reducing environmental impacts, and preserving fungicide efficacy over the long term [169–170].

### *Bacillus*-based products commercialized in Brazil up to 2025 for the control of plant diseases

Table 1 shows the *Bacillus*-based products registered and marketed in Brazil for the control of plant diseases. This table is organized by *Bacillus* species, by mixture of *Bacillus*-based products, and by mixtures of products containing *Bacillus* and antagonistic fungi. For each species or mixture of species, the table provides information on the strains used, the target diseases and pathogens, formulation types, recommended application methods, as well as the corresponding company names and trade names of the products available in the Brazilian market in 2025.

**Table 1:** *Bacillus*-based products commercialized in Brazil up to 2025 for the control of plant diseases

Bacillus species	Strain	Diseases/Pathogens	Formulation	Recommendation	Company	Trademark
*Bacillus amyloliquefaciens*	GNC005A	Anthracnose/*Colletotrichum truncatum*, Target spot/*Corynespora cassiicola*	SC	Foliar application	Gênica Inovação Biotecnológica S.A.	Agathos
*B. amyloliquefaciens*	CBMAI 1301	Anthracnose/*Colletotrichum lindemuthianum*,* Colletotrichum gloeosporioides*	SC	Foliar application	Agrobiológica Soluções Naturais Ltda. Comdeagro - Cooperativa Mista de Desenvolvimento do Agronegócio Dillon Biotecnologia Ltda. Simbiose Biociencias S.A. Bioma Ind. Com. e Distribuição Ltda. Biota Innovations Ind. e Com. de Bioprodutos Ltda.	Amanzi Amitrix Bactel, Duobac Meta, Easy Amylo Biagro Proteção FX Protection, Artefato Velez, Ajaax, Questmyll, Onceres, BRT Protect, Gardeon, BS Inox, Noduvel, Argento, Lorens, Tratus F
*B. amyloliquefaciens*	D-747	Purple blotch/*Alternaria porri*, Gray mold/*Botrytis cinerea*, Postbloom fruit drop /*Colletotrichum acutatum*, Black sigatoka/*Pseudocercospora fijiensis*, Damping-off/*Pythium ultimum*, Rhizoctonia disease/*Rhizoctonia solani*, Powdery mildew/*Sphaerotheca fuliginea*,* Podosphaera fuliginea*,* Erysiphe polygoni*,* Erysiphe necator*, White mold/*Sclerotinia sclerotiorum*, Anthracnose/*C. gloeosporioides*, Alternaria leaf blight/*Alternaria dauci*, Early blight/*Alternaria solani*, Black spot/*Phyllosticta citricarpa*, Bull’s eye rot/*Neofabraea perennans*	SL WG	Foliar application, drench, planting furrow	Mitsui & Co Brasil S.A. Iharabras S.A. Indústrias Química	Amylo-X SL, Bacilo-X Eco-Shot
*B. amyloliquefaciens*	PTA-4838	Root-knot nematode/*Meloidogyne incognita*,* Meloidogyne javanica*, Reniform nematode/*Rotylenchulus reniformis*, Cyst nematode/*Heterodera glycines*, Root lesion nematode/*Pratylenchus brachyurus*,* Pratylenchus zeae*	SC	Seed treatment	Sumitomo Chemical Brasil Ind. Química S.A.	Aveo EZ, Aveo ST, Streat, Lumialza
*B. amyloliquefaciens*	UMAF6614	Root-knot nematode/ *M. incognita*,* M. javanica*,* Meloidogyne paranaensis*,* Meloidogyne exigua*, Root lesion nematode/*P. zeae*,* P. brachyurus*	WP	Seed treatment, planting furrow, drench	Koppert do Brasil Holding S.A.	Boneville, Chevelle, Veraneio
*B. amyloliquefaciens*	MBI600	Bull’s eye rot/*N. perennans*, Leaf spot/*Botrytis squamosa*, Citrus black spot/*P. citricarpa*, Black rot/*Xanthomonas* *campestris*, Powdery mildew/*U. necator*,* S. fuliginea*, Gray mold/*B. cinerea*, Damping-off/*R. solani*, Yellowing, Damping-off/*P. ultimum*, Common scab/*Streptomyces scabiei*, Fusariosis/*Fusarium solani*, Root-knot nematode/*M. incognita*,* M. javanica*, Cyst nematode/*H. glycines*, Root lesion nematode/*P. brachyurus*,* P. zeae*, Reniform nematode/*R. reniformis*	WP SC	Foliar application, drench, planting furrow Seed treatment, planting furrow	Basf S.A. Nufarm do Brasil Ltda.	Duravel Evolvance, Trunemco, Vinemco, Verdiva
*B. amyloliquefaciens*	SIMBI BS 10	Root lesion nematode/*P. brachyurus*, Root-knot nematode/*M. incognita*,* M. exigua*, Cyst nematode/*H. glycines*, White mold/*S. sclerotiorum*	SC	Seed treatment, drench, planting furrow	Simbiose Biociências S.A.	Faciens Protection, Inlayon, Inlayon Eco, NemaControl, NemaControl Super
*B. amyloliquefaciens*	IBSBF 3236	Gray mold/*B. cinerea*	SC	foliar application	Lallemand Soluções Biológicas Ltda.	Lalstop 132 SC
*B. amyloliquefaciens*	BV03	Root lesion nematode/*P. brachyurus*,* P. zeae*, Charcoal rot/*Macrophomina phaseolina*, Root-knot nematode/*M.incognita*,* M. javanica*,* Meloidogyne graminicola*, Spiral nematode/*Helicotylenchus dihystera*, Cyst nematode/*H. glycines*, Stalk rot/*Fusarium verticillioides*	SC	Seed treatment, drench, planting furrow, foliar application	Vittia S.A.	No-Nema, Nema-Attack, Nema-Guard, Bio Baciens, Cropwinner Eco Nemaxy, Vitalforce Bio, Amilofaciens, Bionova Sanus S-Nema, Nematha, Klaatu, Skshield, Glock Nema, SB Rootilis Fresh, Carmelo Life, Protive, BAVTT 0324
*B. amyloliquefaciens*	CL3	Gray mold/*B. cinerea*, Asian soybean rust/*Phakopsora pachyrhizi*, Anthracnose/*C. gloeosporioides*, Fusarium disease/*F. solani*, White spot/*Phyllachora maydis*, Root rot/*P. ultimum*, Areolate mildew/*Ramularia areola*, Damping-off, Rhizoctonia disease /*R. solani*, White mold/*S. sclerotiorum*, Powdery mildew/*S. fuliginea*, Black rot/*Xanthomonas campestris*	SC	Foliar application, planting furrow	Sinon do Brasil Ltda.	Sinsmart CEP CL3, Wanda
*B. amyloliquefaciens*	FZB24	Rhizoctonia disease/*R. solani*, Anthracnose/*C. gloeosporioides*, Powdery mildew/*S. fuliginea*,* U. necator*, Gray mold/*B. cinerea*, White mold/*S. sclerotiorum*, Alternaria leaf blight/*A. solani*	WP	Foliar application, planting furrow	Syngenta Proteção de Cultivos Ltda.	Taegro, Krivesta
*B. amyloliquefaciens*	ENV503	Root lesion nematode/*P. brachyurus*, Damping-off/*R. solani*, White mold*/S. sclerotiorum*, Asian soybean rust/*P. pachyrhizi*	WP	Seed treatment, planting furrow	Lemma Agronegócios Importação e Exportação Ltda.	Companion Maxx WP
*B. amyloliquefaciens*	CBMAI 2356	Root lesion nematode/*P. brachyurus*,* P. zeae*	SC	Seed treatment, planting furrow	Bioma Ind. Com. e Distribuição Ltda.	Nema Protection, Nemax
*B. amyloliquefaciens*	IMA 411	Root lesion nematode/*P. brachyurus*, Root-Knot Nematode/*M. incognita*	SC	Seed treatment, planting furrow	Comdeagro - Cooperativa Mista de Desenvolvimento do Agronegócio	AmyloTrop
*B. amyloliquefaciens*	CN-307	Root lesion nematode/*P. brachyurus*, Root-Knot Nematode/*M. incognita*, Cyst nematode/*H. glycines*	SC	Planting furrow	NOOA Ciência e Tecnologia agrícola Ltda.	Auba, Teros
*B. amyloliquefaciens*	CCT7995	Root lesion nematode/*P. brachyurus*	SC	Planting furrow	Solubio Tecnologias Agrícolas S.A.	Bio Balance, Duobac-Bioagreen, SoluSafe, Now Lock, Glock Nema SC, BCH Biofaciens
*Bacillus firmus (Cytobacillus firmus)*	CCT0227	Root lesion nematode/*P. brachyurus*, Root-knot nematode/*M. javanica*,* M. graminicola*	SC	Seed treatment, planting furrow	Total Biotecnologia Ind. e Com. S.A.	BTP 177 − 21, BTP 177–21 A
*B. firmus (C. firmus)*	I-1582	Root lesion nematode/*P. brachyurus*, Root-knot nematode/*M. incognita*, Cyst nematode/*H. glycines*, Reniform nematode/*R. reniformis*		Seed treatment, planting furrow	Basf S.A.	Andril Prime, Oleaje Prime, Votivo Prime
*Bacillus licheniformis*	S2903	Root lesion nematode/*P. brachyurus*	SC	Planting furrow	Solubio Tecnologias Agrícolas S.A.	Bio Elipso
*Bacillus methylotrophicus*	UFPEDA 20	Root lesion nematode/*P. brachyurus*, Root-Knot Nematode/*M. javanica*	SC	Seed treatment, Foliar application	Lallemand Soluções Biológicas Ltda.	Onix OG
*B. methylotrophicus*	S2908	Root lesion nematode/*P. brachyurus*	SC	Planting furrow, drench	Solubio Tecnologias Agrícolas S.A.	Solu Velezensis
*Bacillus pumilus*	QST 2808	Anthracnose/*C. lindemuthianum*, Purple blotch/*Alternaria porri*, Gray mold/*B. cinerea*, Powdery mildew/*S. fuliginea*,* U. necator* Early blight/*A. solani*, Bull’s eye rot/*N. perennans*	SC	Foliar application	Bayer S.A.	Sonata
*B. pumilus*	S2907	Powdery mildew/*Sphaerotheca macularis*	SC	Foliar application	Solubio Tecnologias Agrícolas S.A.	Solu Shield, Solution Pro Evolution SC, Viva Bio Lorens
*B. pumilus*	CNPSo3203	Septoria brown spot/*Septoria glycines*, Target spot/*C. cassiicola*, Cercospora leaf blight/*Cercospora kikuchii*, Anthracnose/*C. lindemuthianum*,* C. gloeosporioides*, Early blight/*A. solani*, Coffee leaf rust/*Hemileia vastatrix*, Brown rust/*Puccinia melanocephala* Orange rust/*Puccinia kuehnii*, Brown spot/*Cercospora longipes*, Red rot/*Colletotrichum falcatum*, Areolate mildew/*R. areola*, Southern rust/*Puccinia polysora*, Tropical rust/*Physopella zeae*, Common rust/*Puccinia sorghi*, Target spot/*C. cassiicola*, Tar spot/*P. maydis*	SC	Foliar application	Koppert do Brasil Holding S.A.	Caravan, Grabber, Grabber by Koppert, BTP 005–19
*Bacillus subtilis*	QST 713	Anthracnose/*C. gloeosporioides*,* C. acutatum*, Fusarium wilt*/F. oxysporum* f.sp. *lycopersici*, Bacterial Leaf streak/*Xanthomonas vasicola* pv. *vasculorum*, Bull’s eye rot/*N. perennans*, Root rot/*P. ultimum*, Alternaria leaf blight*/Alternaria dauci*, Gray mold/*B. cinerea*, Purple blotch*/Alternaria porri*, Powdery mildew*/Sphaerotheca macularis*, Black sigatoka*/P. fijiensis*, Powdery mildew/*S. fuliginea*, Rhizoctonia disease*/R. solani*, Bacterial spot*/X. vesicatoria*, Citrus canker/*Xanthomonas citri* subsp. *citri* Common scab*/Streptomyces scabiei*, Root-knot nematode*/M. incognita*,* M. javanica*, Root lesion nematode/*P. brachyurus*, White mold/*S. sclerotiorum*	SC	Foliar application, drench	Bayer S.A.	Serenade
*B. subtilis*	Y1336	Root lesion nematode/*P. zeae*, Root-knot nematode/*M. incognita*, Citrus canker/*X. citri* subsp. c*itri*, Bacterial wilt/*Ralstonia solanacearum*, Black rot/*X. campestris* pv. *vesicatoria*, Rhizoctonia disease/*R. solani*, Bull’s-eye rot/*N. perennans*, Purple blotch/*A. porri*, Coffee leaf rust/*H. vastatrix*, Gray mold/*B. cinerea*	WP	Foliar application, drench, planting furrow	UPL do Brasil Ind. e Com. De Insumos Agropecuários S.A.	Animus, Tacap
*B. subtilis*	UFPEDA 764	Root lesion nematode/*P. brachyurus*, Root-knot nematode/*M. javanica*	SC	Seed treatment, foliar application	Lallemand Soluções Biológica Ltda.	Rizos, Rizos OG
*B. subtilis*	CCT 1331	Root-knot nematode/*M. incognita*	GL	Foliar application, planting furrow, drench	TZ Biotec Ltda.	Subtillyd FR 25
*B. subtilis*	CCTB04	Root lesion nematode/*P. brachyurus*,* P. zeae*, Root-knot nematode/*M. incognita*	SC	Seed treatment, Planting furrow, drench	Total Biotecnologia Ind. e Com. S.A.	BTP 002-18AB, Pusher, Vult Plus
*B. subtilis*	CCT 7993	Root lesion nematode/*P. brachyurus*, Root-knot nematode/*M. javanica*	SC	Planting furrow, drench	Solubio Tecnologias Agrícolas S.A.	SoluBio Raiz Performance, Raiz Protect, Now Guard, Farpado
*B. subtilis*	Bk-Bs01	Dry root rot/*F. solani*, Damping-off, Rhizoctonia disease/*R. solani*, Anthracnose/*C. gloeosporioides*	SC	Planting furrow, foliar application	Mezfer BR Soluções Agrícolas Ltda.	Astor, Baktillis
*B. subtilis*	CNPSo 2657	Root lesion nematode/*P. brachyurus*, Root-knot nematode/*M. incognita*,* M. javanica*, Cyst nematode*/H. glycines*, Damping-off/*R. solani*, White mold/*S. sclerotiorum*	SC	Seed treatment, planting furrow,	Total Biotecnologia Ind. e Com. S.A.	Bioessence Drive, Furatrop, Nematrop, Biotrinsic, Nemafree, Submax, Nemaster, Paladyo
*B. subtilis*	IAB/BS03	Late blight/*Phytophthora infestans*, Downy mildew/*Pseudoperonospora cubensis* Coffee leaf rust/*H. vastatrix*, Asian soybean rust/*P. pachyrhizi*, Target spot/*C. cassiicola*, Septoria brown spot/*S. glycines*, Anthracnose/*C. lindemuthianum* Powdery mildew/*Leveillula taurica*,* S. fuliginea*,* U. necator*	EC WP WP	Foliar application	Tradecorp do Brasil Com. de Insumos Agrícolas Ltda.	Milarum Row-Vispo Ospo Vi55
*B. subtilis*	ATCC 6051	Root-knot nematode/*M. incognita*	WP	Planting furrow, drench	Improcrop do Brasil Ltda.	RELI3VER
*Bacillus thuringiensis*	RTI545	Cyst nematode/*H. glycines*, Reniform nematode/*R. reniformis*	SC	Seed treatment	FMC Química do Brasil Ltda.	Avizon, Avizon BR, Rovisar, Rovisar BR
*Bacillus velezensis*	CCT 7943	Root lesion nematode/*P. brachyurus*, Root-knot nematode/*M. incognita*	SC	Seed treatment	Bioma Ind. Com e Distribuição Ltda.	Biagro Raiz, Nerut, Bioma – B.Bv 10
*B. velezensis*	Labim 40	Anthracnose/*C. truncatum*, Target spot/*C. cassiicola*, White spot/*Pantoea ananatis*, Septoria brown spot/*Septoria glycines*	SC	Foliar application, planting furrow	Simbiose Biociencias S.A.	FrontierControl
*B. velezensis*	ASN09	Root lesion nematode/*P. brachyurus*, Root-knot nematode/*M. incognita*, Root rot, Damping-off/*R. solani*	SC	Seed treatment	Agrobiológica Soluções Naturais Ltda.	Shieldshock, Amylotrix
*B. velezensis*	FZB42	Root lesion nematode/*P. brachyurus*, Root rot/*R. solani*	SC	Seed treatment, planting furrow.	Andermatt do Brasil Soluções Biológicas Ltda.	Nembac 42, Amyprotec 42
*B. velezensis*	IBSBF 3238	Root lesion nematode/*P. brachyurus*, Root-Knot Nematode/*M. javanica*	SC	Foliar application, seed treatment	Lallemand Soluções Biológicas Ltda	Lalnix Over SC
*B. velezensis*	CCTB09	Root-knot nematode/*M. incognita*, Fusarium wilt/*F. solani*	SC	Seed treatment,	Total Biotecnologia Ind. e Com. S.A.	DEF-MIC-089-24
*B. velezensis*	CNPSo 3602	Root-knot nematode/*M. incognita*,* M. javanica*, Reniform nematode/*R. reniformis*, Cyst nematode/*H. glycines*, Root lesion nematode/*P. brachyurus*, Fusarium wilt/*F. solani* Damping-off/*R. solani*, Charcoal rot/*M. phaseolina* Root-knot nematode/*M. incognita*, Fusarium wilt/*F. solani*	SC	Seed treatment, planting furrow, drench, foliar application Seed treatment	Syngenta Proteção de Cultivos Ltda. Total Biotecnologia Ind. e Com. S.A.	Arvatico, Certano BTP 007–19
*B. velezensis*	BV09	Root lesion nematode/*P. zeae*, Root-knot nematode/*M. incognita*,* M. javanica*,* M. exigua*,* M. paranaensis*, Fusarium wilt/*F. oxysporum*	SC	Foliar application, planting furrow.	Vittia S.A.	Biobaci, Baci-Attack, Baci-Guard, Vitalforce Bio Phako, Cropwinner Eco Vastax
*B. velezensis*	BV02	Anthracnose/*C. gloeosporioides*,* C. lindemuthianum*,* C. truncatum*, Coffee leaf rust/*H. vastatrix*, Powdery mildew/*U. necator*, Black rot/*X. vesicatoria*, Alternaria leaf blight/*A. solani*, Postbloom fruit drop/*C. acutatum*, Bacterial speck/*Pseudomonas syringae*, Bacterial halo blight*/Pseudomonas syringae* pv. *garcae*, Asian soybean rust/*P. pachyrhizi*, White mold/*S. sclerotiorum*, Target spot/*C. cassiicola* Gray mold/*B. cinerea*, White spot/*P. maydis*, Areolate mildew/*R. areola*, Phoma leaf spot/*Phoma costaricensis*, Brown spot /*Cercospora coffeicola*	SC	Foliar application	Vittia S.A.	Bio-imune, Multi-Attack, Multi-Guard, FungiOuro, Biologic Aerum, Brandt Clearfol, Power Bac Inductor, SKDefense, Solution Pro Evolution, BioAgross Andorinha
*B. velezensis*	CECT8237	Cercospora leaf blight/*Cercospora kikuchii*, Brown spot/*Cercospora longipes*, Brown spot/*Cercospora zeae-maydis*, Septoria brown spot/*Septoria glycines* Target spot/*C. cassiicola*, Red rot*/C. falcatum*, White spot/*P. maydis*, Tropical rust/*Physopella zeae*, Orange rust/*Puccinia kuehnii*, Brown rust/*Puccinia melanocephala*, Southern rust/*Puccinia polysora*, Common rust/*Puccinia sorghi*, Asian soybean rust/*P. pachyrhizi*	SC	Foliar application, planting furrow, drench	Koppert do Brasil Holding S.A.	Macan
*Priestia megaterium*	CCT7987	Root lesion nematode/*P. zeae*	SC	Planting furrow, seed treatment	Solubio Tecnologias Agrícolas S.A.	Bio Solubilize, Phós Bio, Now Active, Agripon Phosbacter Nema
*P. megaterium*	CCT1623	Root-knot nematode/*M. incognita*	GL	Planting furrow	TZ Biotec Ltda.	Megateryd FR25
**Consortium with** ***Bacillus*** **spp.**						
*B. subtilis* *B. subtilis*	CBMAI1680CNPSo 2657	Root-knot nematode/*M. incognita*	SC	Foliar application,	Total Biotecnologia Ind. e Com. S.A.	BTP 500 − 21 BTP 500–21 A
*B. velezensis* *B. velezensis*	CBMAI 1304 CBMAI 1301	Anthracnose/*C. gloeosporioides*,* C. lindemuthianum*,* C. truncatum*, Red stripe/*Paracidovorax avenae*, Brown spot /*Cercospora coffeicola*, Target spot/*C. cassiicola*, Aschochyta leaf blight/*Ascochyta coffeae*, White spot/*P. maydis*, Phoma leaf spot/*Phoma costaricensis*, Areolate mildew/*R. areola*	SC	Foliar application, planting furrow, drench	Massen Produtos Biológicos S.A. Agrivalle Brasil Ind. e Com. de Produtos Agrícolas S.A.	Restrict, Vitanica Duo Protect, Zigurat, BioDobl, Amosbio, Wixx, Accuracy, Agtecmmon Twixx-A
*B. amyloliquefaciens* *B. velezensis*	CCT8135 CCT8136	Gray mold/*Botrytis cinerea*, Asian soybean rust/*P. pachyrhizi*, Target spot/*C. cassiicola*	SC	Foliar application	Bem da Terra Biológicos Ltda. Nitro Química Brasileira	Proteva, Dualbac, Onceres Plus, Multivex Égide, Égide Max
*B. amyloliquefaciens* *B. subtilis*	BAC105 BAC116	Root lesion nematode/*P. brachyurus*, Cyst nematode/*H. glycines*, White mold/*S. sclerotiorum*	WP	Foliar application, seed treatment, planting furrow	Superbac Biotechnology Solutions S.A.	Tilsis
*B. amyloliquefaciens* *B. subtilis*	BAC105 BAC097	Root lesion, nematode/*P. brachyurus*,* P. zeae*, Cyst nematode/*H. glycines*, Root-knot nematode/*M. incognita*	WP	Planting furrow, drench	Superbac Biotechnology Solutions S.A.	SBAC8
*B. subtilis* *B. velezensis*	BAC004 BAC079	Root-knot nematode/*M. incognita*, Damping-off/*R. solani*, Root lesion nematode/*P. zeae*	WP	Planting furrow	Superbac Biotechnology Solutions S.A.	SBAC6
*B. subtilis* *B. subtilis*	BAC059 BAC116	Damping-off/*R. solani*, White mold/*S. sclerotiorum*, Dry root rot/*F. solani*	SC	Foliar application, seed treatment	Superbac Biotechnology Solutions S.A.	Superguard Liq
*B. thuringiensis* *B. thuringiensis*	N3 Anemophila	Root-knot nematode/*M. javanica*	WP	Furrow planting	Bio Insumos Nativa do Brasil Ltda.	Bafex
*B. subtilis* *B. pumilus*	BAC059 BAC114	Damping-off/*R. solani*	WP	Planting furrow	Superbac Biotechnology Solutions S.A.	Superguard Pro
*B. velezensis* *B. subtilis*	ORN14 ORN160	Root lesion nematode/*P. brachyurus*, Root-knot nematode/*M. javanica*	SC	Seed treatment	Adama Brasil S.A.	ADA FM 267/21
*B. subtilis* *B. velezensis*	ESALQ-EpD2-5, ESALQ-RZ1MS9	Root lesion nematode/*P. brachyurus*, Root-Knot Nematode/*M. incognita*,* M. javanica*, Charcoal rot/*M. phaseolina*, Fusarium wilt/*F. solani* f. sp. *phaseoli*	SC	Seed treatment, planting furrow	Bionat Soluções Biológicas Ltda.	Peregrino, Bio Pegasus, Canário, Glock Nema
*B. velezensis* *Paenibacillus ottowii*	BRM046334 BRM05345	Fusarium wilt//*Fusarium pallidoroseum*, Stalk rot*/Fusarium verticillioides*	SC	Seed treatment	Simbiose Biociências S.A.	EficazControl
*B. velezensis* *B. subtilis*	RTI301 RTI477	Anthracnose/*C. dematium*,* C. gloeosporioides*,* C. lindemuthianum*,* C. truncatum*, Stem rot/*Pythium aphanidermatum* Damping-off/*R. solani*, Fusarium wilt/*F. oxysporum* f.sp. *vasinfectum*, *F. oxysporum* f. sp. *phaseoli* Yellowing/*P. ultimum*, Blast/*Pyricularia oryzae*, Citrus canker/*X. citri* subsp. c*itri*, Gummy stem blight/*Didymella bryoniae*, Early leaf spot/*Cercospora arachidicola*, Target spot/*C. cassiicola*, Bacterial spot/*Xanthomonas vesicatoria*, Septoria brown spot/*S. glycines*, White mold/*S. sclerotiorum*, Areolate mildew/*Ramulariopsis pseudoglycines*, Black Sigatoka/*P. fijiensis*	SC	Seed treatment, foliar application, drench, planting furrow	FMC Química do Brasil Ltda.	Ataplan, Aratel, Provilar, Kinevar
*B. subtilis* *B. velezensis*	BS34 BV59	Seed decay/*Diaporthe longicolla*, Red stripe/*Paracidovorax avenae*, Early Blight/*A. solani*, Areolate mildew/*R. areola*, Coffee leaf rust/*H.vastatrix*, White spot/*P. maydis*, Fusarium head blight/*Fusarium graminearum*	SC	Foliar application, drench, planting furrow	Genica Inovação Biotecnológica S.A.	GNC014
*B. subtilis* *B. licheniformis*	FMCH002 FMCH001	Root lesion nematode/*P. brachyurus. P. zeae*, Root-knot nematode/*M. incognita*,* M. graminicola*,* M. javanica*,* M. exigua*, Cyst nematode/*H. glycines*	WS	Seed treatment, planting furrow	FMC Química do Brasil Ltda	Presence, Presence Flex, Kalivar, Presence Full, Fortmax. Quartzo, Surface
*B. subtilis* *B. licheniformis*	BAC059 BAC112	Root-knot nematode/*M. incognita*	WP	Seed treatment, Planting furrow	Superbac Biotechnology Solutions S.A.	Tillez, Zenzis
*Bacillus paralicheniformis* *B. paralicheniformis* *Bacillus subtilis*	CH2970CH0273 CH4000	Root lesion nematode/*P. brachyurus*,* P. zeae*, Root-knot nematode/*M. incognita*, Cyst nematode/*H. glycines*, Reniform nematode/*R. reniformis*	SC	Planting furrow	UPL do Brasil Ind. e Com. de Insumos Agropecuários S.A.	Nimaxxa, Unnat
*B. subtilis* *B. velezensis* *B. pumilus*	SVG00045-B SVG00047-B SVG00046-B	Septoria brown spot/*S. glycines* Cercospora leaf blight/*C. kikuchii*, Target spot/*C. cassiicola*, Brown spot/*Cercospora zeae-maydis*, Late leaf spot/*Cercosporidium personatum*, Postbloom fruit drop/*C. acutatum*, Northern corn leaf blight/*Exserohilum turcicum*, White mold/*S. sclerotiorum*, Red rot/*C. falcatum*, Areolate mildew/*R. areola*, Stalk rot/*Stenocarpella maydis*, Coffee leaf rust/*H. vastatrix*, Powdery mildew/*S. fuliginea*,* Blumeria graminis*,* Sphaerotheca pannosa*, Anthracnose/*C. lindemuthianum*,* C. truncatum*,* C. gloeosporioides*, Leaf rust/*Puccinia triticina*, Tan spot/*Drechslera tritici-repentis*, Net blotch/*Drechslera teres*, Southern rust/*Puccinia polysora*, Scab/*F. graminearum*, Leaf rust/*Puccinia hordei*, Tar spot/*P. maydis*, Blast/*P. oryzae*, Gray mold/*B. cinerea*, Black Sigatoka/*P. fijiensis*, Southern blight/*Agroathelia rolfsii*	SC	Foliar application	Syngenta Proteção de Cultivos Ltda.	Reverb
*Bacillus inaquosorum* *B. licheniformis* *B. velezensis*	CBMAI2783 CBMAI2779 CBMAI2781	Root lesion nematode/*P. brachyurus*	SC	Planting furrow	Inflora Biociência Ltda.	Cordel, Exclusiv EVO
*B. licheniformis* *B. velezensis* *P. megaterium*	CBMAI2639 CBMAI2618 CBMAI2940	Root lesion nematode/*P. brachyurus*	SC	Planting furrow	Gênica Inovação Biotecnológica S.A.	GNC013
*B. subtilis* *B. licheniformis* *Brevibacillus parabrevis*	N5 Copihue N4	White mold/*S. sclerotiorum*, Early blight/*A. solani*	WP	Drench, foliar application	Bio Insumos Nativa do Brasil Ltda.	Nacillus Max
*B. subtilis* *B. velezensis* *B. pumilus*	CNPSo2720 CNPSo3602 CNPSo3202	Septoria brown spot/*S. glycines*, Citrus post-bloom/*C. acutatum*, Powdery mildew/*S. fuliginea*, Gray mold/*B. cinerea*, White mold/*S.sclerotiorum*, Black Sigatoka/*P. fijiensis*, Southern blight/ *Agroathelia rolfsii*, Anthracnose/*C. gloeosporioides*	SC	Foliar applications	Total Biotecnologia Ind. e Com. S.A.	Bioshield
*B. subtilis* *B. velezensis* *B. pumilus*	CCTB04 CCTB09 CCTB05	Septoria brown spot/*S. glycines*, Cercospora leaf blight/*C. kikuchii*, Target spot/*C. cassiicola*, Gray leaf spot/*C. zeae-maydis*, Late leaf spot/*Cercosporidium personatum*, Postbloom fruit drop/*C. acutatum*, Northern corn leaf blight/*Exserohilum turcicum*, White mold/*S. sclerotiorum*, Red rot/*C. falcatum*, Areolate mildew /*R. areola*, Stalk rot/*Stenocarpella maydis*, Coffee leaf rust/*H. vastatrix*, Powdery mildew/*S. fuliginea*,* Blumeria graminis*,* S. pannosa*, Anthracnose/*C. lindemuthianum*,* C. truncatum*,* C. gloeosporioides*, Leaf rust/*Puccinia triticina*, Tan spot/ *Drechslera tritici-repentis*, Net blotch/ *Drechslera teres*, Southern rust/*Puccinia polysora*, Scab/*F. graminearum*, Leaf rust/*Puccinia hordei*, Tar spot/ *P*. *maydis*, Blast/*P. oryzae*, Gray mold/*B. cinerea*, Black Sigatoka/*M. fijiensis*, Southern blight/*Agroathelia rolfsii*, Anthracnose/*C. gloeosporioides*, Alternaria disease/*Alternaria alternata*, Leaf stripe/*Paracidovorax avenae*	SC	Foliar applications	Total Biotecnologia Ind. e Com. S.A.	Bombardeiro, Lastro
*B. pumilus* *B. velezensis* *B. velezensis*	DC61 DC81 DC88	Root rot/*R. solani*, Early blight/*A. solani*, Red rot/*C. falcatum*, Anthracnose/*C. lindemuthianum*, Gray mold/*B. cinerea*	SC	Foliar application, drench, planting furrow	Apoena Biosoluções do Brasil Ltda.	Forblend FS Premium, Bioultra-X, RedLock
*Priestia aryabhattai* *Bacillus haynesii* *Niallia circulans*	CNMAI 1120 CCT7926 CCT0026	Growth promoter	SC	Seed, planting furrow, foliar application	Total Biotecnologia Ind. e Com. S.A.	Bioasis Power, Biolord,
*B. amyloliquefaciens* *B. velezensis* *B. thuringiensis*	CNPSo 3202 CNPSo 3602 CNPSo 3915	Damping-off/*R. solani*, Root lesion nematode/*P. brachyurus*,* P. zeae*, Black rot/*Ceratocystis paradoxa*, Charcoal rot /*M. phaseolina*, Root-knot nematode/*M. incognita*,* M. javanica*,* M. exigua*,* M. graminicola*, Reniform nematode/*R. reniformis*, Cyst nematode/*H. glycines*, Dry root rot/*Fusarium solani* f. sp. *phaseoli*, Southern blight/*Agroathelia rolfsii*	SC	Seed treatment, drench, planting furrow, foliar application	Total Biotecnologia Indústria e Comércio S.A. Adama Brasil S.A.	Biomagno, Bioharmer, BTP 011–19 Protege
*B. amyloliquefaciens* *B. amyloliquefaciens* *B. subtilis* *B. subtilis*	BAC076 BAC077 BAC059 BAC116	Fusarium wilt/*F. oxysporum*, Damping-off, Rhizoctonia disease /*R. solani*, White mold/*S. sclerotiorum*, Asian soybean rust/*P. pachyrhizi*, Dry root rot/*F. solani*, Target spot/*C. cassiicola*	WP	Foliar application, Planting furrow, drench	Superbac Biotechnology Solutions S.A.	Superguard
*B. amyloliquefaciens* *B. amyloliquefaciens* *B. subtilis* *B. thuringiensis*	SVG 00027-B SVG 00028-B SVG 00030-B SVG 00029-B	Root-knot nematode/*M. incognita*	SC	Seed treatment, planting furrow	Biota Innovations Ind. e Com. de Bioprodutos Ltda Comdeagro - Cooperativa Mista de Desenvolvimento do Agronegócio	Bamytis, Royalprezor, Basuty, Exclusiv, Batynem, Bany, Onprotect N, BRT Block, Bacill Mix, BS Aquanema, Bioboost, Cropbio Nema, Nodubionema, Bunker 4, Biocaz Robust, Baker 4, Bio Titan Plus NemaZak
*B. amyloliquefaciens* *B. licheniformis* *B. subtilis* *P. megaterium*	CCT 8104 ATCC 14,580 ATCC 6051 CCT8101	Root-Knot Nematode/*M. incognita*	SC	Drench, planting furrow	Companhia Nitro Química Brasileira	Elmo Max, NT 5–24
*B. amyloliquefaciens* *B. subtilis* *B. licheniformis* *B. velezensis*	BAC105 BAC004 BAC112 BAC079	Root lesion nematode/*P. brachyurus*,* P. zeae*, Root-Knot Nematode/*M. incognita*,* M javanica*, Cyst nematode/*H. glycines*	WP	Seed treatment	Superbac Biotechnology Solutions S.A.	Supershield, Supershield Liq, SBAC10
*Paenibacillus azotofixans* *B. subtilis* *B. licheniformis* *B. circulans*	CCT4719 CCTB04 CCTB07 CCT0026	Damping-off/*R. solani.*, Root lesion nematode/*P. brachyurus*, Black rot /*Ceratocystis paradoxa*, Charcoal rot/*M. phaseolina*, Root-knot nematode/*M. incognita*,* M. javanica*,* M. exigua*, Burrowing nematode/*Radopholus similis*, Cyst nematode/*H. glycines*, White mold/*S. sclerotiorum*	SC	Seed, planting furrow, foliar application	Total Biotecnologia Indústria e Comércio S.A.	Biolucro, BTP 167–21 A
*B. amyloliquefaciens* *B. subtilis* *B. licheniformis* *B. velezensis* *B. pumilus*	Ba30 Bs34 GNC004A GNC004C BPU	Root lesion nematode/*P. brachyurus*, Fusarium wilt/*F. oxysporum*	SC	Drench, planting furrow	Gênica Inovação Biotecnológica S.A.	GNC004C
*B. amyloliquefaciens* *B. licheniformis* *B. subtilis* *B. subtilis* *B. velezensis* *P. megaterium*	BA30 GNC004A BS34 BSS GNC004C PM60	Fusarium wilt/*F. oxysporum*, Root lesion nematode/*P. brachyurus*	SC	Seed treatment, planting furrow	Gênica Inovação Tecnológica S.A.	Dulia Pro GNC004C
**Consortium with fungi**						
*B. amyloliquefaciens* *Trichoderma asperellum*	IMA412 URM8117	Root rot/*R. solani*	WG	Seed treatment	Comdeagro – Coop. Mista de Desenvolvimento do Agronegócio	Aspemax
*B. subtilis* *Trichoderma harzianum*	CCT 0480 CCT2160	Root-knot nematode/*M. incognita*	SC	Planting furrow	Vital Brasil Chemical Ind. e Com. Prod. Químicos Ltda	Bionmt, Econema
*B. amyloliquefaciens* *T. asperellum* *T. harzianum*	CCT 7901 URM 8120 URM 8119	Anthracnose/*C. lindemuthianum*, Damping-off/*R. solani*, White mold/*S. sclerotiorum*, Root lesion nematode/*P. brachyurus*, Black rot/*C. paradoxa*, Charcoal rot/*M. phaseolina*	WG	Foliar application, planting furrow	FMC Química do Brasil Ltda Ballagro AgroTecnologia Ltda.	Cablar Pardella, Primitivo, Ourotricx, Soil Safe
*B. velezensis* *B. velezensis* *T. harzianum*	CBMAI 1301 CBMAI 1304 CPQBA 040-11DRM 09	Damping-off, Rhizoctonia disease/*R.solani*, White mold/*S. sclerotiorum*, Charcoal rot/*M.phaseolina*, Black rot/*C. paradoxa*, Fusarium wilt/*F. solani*	WP	Seed treatment, drench, planting furrow	Agrivalle Brasil Ind. e Com. de Prod. Agrícolas S.A.	Shocker, Shocker-A
*B. amyloliquefaciens* *T. asperellum* *T. harzianum*	CCT 7901 URM 8120 URM 8119	Anthracnose/*C. lindemuthianum*, White mold/*S. sclerotiorum*, Damping-off, Rhizoctonia disease/*R. solani*	WG	Foliar application, planting furrow, drench	Biota Innovations Ind. e Com. de Bioprodutos Ltda	Tanus, Tricozak
*B. velezensis* *B. velezensis* *T. harzianum*	CBMAI 1301 CBMAI 1304 CPQBA 040-11DRM 09	Damping-off, Rhizoctonia disease/*R. solani*, White mold/*S. sclerotiorum*	WP	Foliar application, planting furrow, drench	Massen Produtos Biológicos S.A.	Torpeno, Trichosmart, Bellator, Bionativus, Biofence, Praky
*B. subtilis* *B. licheniformis* *Purpureocillium lilacinum*	ATCC 6051 ATCC 12,713 CPQBA 040 − 11	Root lesion nematode/*P. brachyurus*, Root-knot nematode/*M. incognita*,* M. exigua*, Cyst nematode/*H. glycines*	WP	Seed treatment, drench, planting furrow	Massen Produtos Biológicos S.A. Agrivalle Brasil Ind. e Com. de Produtos Agrícolas S.A.	Agdommon, Nemaoff, Bionexus, Volga, Fazen, Messenger Profix-A
*B. licheniformis* *B. subtilis* *T. asperellum* *P. lilacinum*	GNC004A Bs34 CBMAI 1622 PL	Root lesion nematode/*P. brachyurus*, Fusarium wilt/*F. oxysporum*	WP	Seed treatment, planting furrow	Gênica Inovação Tecnológica S.A.	Rifle
*B. subtilis* *B. pumilus* *B. velezensis* *T. harzianum*	CCT8004 CCT8005 CCT8003 CCT8009	Target spot/*C. cassiicola*, Root-knot nematode/*M. javanica*	EC	Foliar application, seed treatment	Serquibio Biotecnologia Agrícola Ltda.	BioProctegeh, Cropbio BioSecurity Biosollus, Shelby, Cropbio BioHellmax, Bio-Four
*B. amyloliquefaciens* *B. subtilis* *T. harzianum* *T. asperellum*	SVG-00036B SVG-00037-B SVG-00003-F SVG-00124-F	Damping-off, Rhizoctonia disease/ *R. solani*, White mold/*S. sclerotiorum*	WG	Planting furrow, foliar application	Biota Innovations Ind. e Com. de Bioprodutos Ltda.	Tryba

*SC* Suspension Concentrates, *WG* Water-dispersible Granule, *SL* Soluble concentrates, *WP* Wettable Powder, *WS* Water-dispersible powders for Slurry seed treatment, *EC* Emulsifiable Concentrate, *GL* emulsifiable gel

Fonte: https://agrofit.agricultura.gov.br/agrofit_cons/principal_agrofit_cons

As of 2025, 297 commercial products based on *Bacillus* were registered and marketed, comprising 126 strains of the following species as active ingredients: *Bacillus subtilis* (31 strains), *Bacillus amyloliquefaciens* 27 strains), *Bacillus velezensis* (25 strains), *Bacillus licheniformis* (10 strains), *Bacillus pumilus* (10 strains), *Bacillus thuringiensis* (5 strains), *Bacillus methylotrophicus* (2 strain), *Priestia megaterium* (2 strains), *Bacillus paralicheniformis* (2 strains), *Cytobacillus firmus* (2 strains), *Niallia circulans* (1 strain), *Bacillus haynesii* (1 strain), *Priestia aryabhattai* (1 strain), *Bacillus inquosorum* (1 strain), *Brevibacillus parabrevis* (1 strain), *Paenibacillus azotofixans* (1 strain), *Paenibacillus ottowii* (1 strain) and *Priestia megaterium* (3 strain). In addition, mixed formulation containing fungal biocontrol agents plus *Bacillus* spp. include five strains of *Trichoderma harzianum*, three strains of *Trichoderma asperellum*, and two strains of *Paecilomyces lilacinus* were observed (Table 1).

Among the registered products, 42 companies hold registrations for *Bacillus-*based products. These products are recommended for application via seed treatment, foliar spraying, soil drenching, and planting furrow. The main formulations types include: SC = suspension concentrates, WG = water-dispersible granule, SL = soluble concentrates, WP = wettable powders, WS = water-dispersible powders for slurry seed treatment, GL = emulsifiable gel, and EC = emulsifiable concentrate (Table 1).

*Bacillus*-based products are primarily recommended for the control of the following diseases: root-lesion nematodes, root-knot nematodes, anthracnose, white mold, gray mold, target spot, damping-off, powdery mildew, and cyst nematodes (Table 1).

### Methodology to obtain information on the potential area treated and turnover of *Bacillus-*based products for plant disease control in Brazil in the 2024/2025 crop season

The market panel methodology involves structured, face-to-face interviews conducted with farmers and managers of agricultural properties. The survey covers the main crops in Brazilian agriculture, encompassing the most important regions in terms of cultivated area and production volume. Over the past six crop seasons, Kynetec has conducted approximately 16,000 interviews across different crops, with the sampling designed to consider both regional representativeness and landholding stratification within each region. The data obtained are analyzed using IBM SPSS Statistics software for statistical analysis, data mining, and predictive modeling, which is widely used in the social sciences, market research, and academia (https://www.ibm.com/br-pt/products/spss-statistics).

During the 2019/2020, 2020/2021, 2021/2022, 2022/2023, 2023/2024, and 2024/2025 crop seasons, 15,404; 15,063; 14,839; 15,834; 15,903; and 15,708 interviews were conducted, respectively. Of this total number of interviewees, 30%, 36%, 39%, 43%, 48% and 52% adopted the use of biocontrol agents in the management of diseases and pests, respectively. Therefore, the surveys follow a rigorous statistical process for quantitative research, ensuring high levels of reliability of results and analyses. For each crop, the sample size and the corresponding margins of error are determined based on the indicators and the profile of the investigated universe. Table 2 shows the sampling data and margins of error for the crops surveyed in Brazil during the 2024/2025 crop season.

**Table 2:** Sampling data and margins of error for the crops surveyed in Brazil in the 2024/25 crop season

Crop	Number of interview	Margin of error
Soybean	3,728	1.61%
Fruits and vegetables	2,536	1.95%
Maize (second crop)	2,200	2.09%
Maize (summer crop)	2,004	2.19%
Pasture	1,149	2.89%
Coffee	1,104	2.95%
Dry bean	688	3.74%
Wheat	596	4.01%
Sugarcane	488	4.44%
Irrigated rice	374	5.07%
Citrus	368	5.11%
Sorghum	353	5.22%
Cotton	276	2.40%
Peanut	112	9.26%
Reforestation	84	10.69%
Total	**16**,**060**	

During the interviews, respondents are questioned about the entire phytosanitary management system adopted for each specific crop, encompassing all stages, from seed and soil treatments, to foliar applications and post-harvest practices.

The primary objective of this study is to map the phytosanitary management practices implemented in each production system for each crop season. Based on these data, it is possible to consolidate and characterize the Brazilian market for crop protection products, including agrochemicals and biological agents, at the end-user level.

### Growth trends in the potential area treated of *Bacillus*-based products for plant disease control in Brazil between 2020 and 2025

The total agricultural area in Brazil was 78,564,000; 82,860,000; 88,173,000; 91,391,000; 92,571,000 and 96,102,000 hectares, for the years 2020, 2021, 2022, 2023, 2024, and 2025, respectively. These areas were primarily cultivated with soybean, corn, sugarcane, wheat, coffee, cotton, rice, common bean, citrus, eucalyptus, among other crops. The potential area treated (PAT) with crop protection products (both chemical and biological) was 1,511,064,000; 1,631,818,000; 1,770,877,000; 1,980,006,000; 2,200,268,000, and 2,480,015, 000 hectares from 2020 to 2025, respectively. This represents a 12% annual increase in the potential area treated between 2024 and 2025 (Table 3).

**Table 3:** Total agricultural land (in hectare); total potential area treated (PAT) (in hectare) with crop protection, with biological control-based products, and with *Bacillus-*based products; and total of turnover (millions of US dollar) with crop protection, with biological control-based products, and with *Bacillus-*based products in Brazilian agricultures from 2020 to 2025

Year	2020	2021	2022	2023	2024	2025
Total agricultural land (in ha)	78,564,000	82,860,000	88,173,000	91,391,000	92,571,000	96,102,000
Total of PAT (hectare) with crop protection (chemical + biological control)	1,511,064,000	1,631,818,000	1,770,877,000	1,980,006,000	2,200,268,000	2,480,015,000
Total of PAT (hectare) with biological control agents-based products	22,186,000	26,632,000	42,549,000	52,056,000	65,724,000	86,937,000
Total of PAT (hectare) with *Bacillus-*based products	7,576,610	9,591,760	14,101,930	18,097,660	25,991,710	37,194,240
Total of turnover (x millions of US dollar) with crop protection	13,775	12,510	15,711	21,364	19,915	18,395
Total of turnover (x millions of US dollar) with biological control-based products	271.63	291.89	469.26	697.32	784.41	855.68
Total of turnover (x millions of US dollar) with *Bacillus*-based products	114.31	118.30	186.72	290.12	362.02	403.17

The potential area treated with products containing biological control agents for crop protection in Brazil was 22,186,000; 26,632,000; 42,549,000; 52,056,000; 65,724,000; and 86,937,000 hectares, while the PAT with *Bacillus*-based products was 7,576,610; 9,591,760; 14,101,930; 18,097,660; 25,991,710; and 37,194,240 hectares, for the years 2020, 2021, 2022, 2023, 2024, and 2025, respectively. Thus, the PAT with *Bacillus*-based products accounted for 34%, 36%, 34%, 35%, 40%, and 43% of the total PAT with products containing biological control agents in 2020, 2021, 2022, 2023, 2024, and 2025, respectively (Table 4). These figures indicate increases of 32%, and 35% in PAT between 2024 and 2025 for the products containing biocontrol agents and for *Bacillus-*based products, respectively.

**Table 4:** Potential area treated (PAT) with *Bacillus* species and with total of biological control agents based-products in Brazilian agriculture from 2020 to 2025 (in hectare)

Active ingredient - One species of Bacillus	2020	2021	2022	2023	2024	2025
*Bacillus amyloliquefaciens*	1,129,450	1,697,690	2,379,490	4,364,850	9,704,240	13,871,490
*Bacillus subtilis*	1,949,590	1,971,020	3,265,520	3,132,940	3,387,100	3,352,440
*Bacillus pumilus*	177,930	93,710	258,880	512,740	1,164,140	2,861,570
*Bacillus firmus (Cytobacillus firmus)*	34,170	685,980	713,490	1,378,100	1,770,830	1,624,020
*Bacillus velezensis*	900	117,310	240,450	288,770	880,520	1,598,270
*Bacillus methylotrophicus*	770,760	1,086,580	1,493,540	1,656,930	1,695,830	1,591,680
*Priestia megaterium*						2,100
*Bacillus licheniformis*						13,370
*Bacillus* sp.	398,190	324,760	118,380	108,000	1,110	
*Bacillus thuringiensis*	1,951,370	1,540,350	1,869,110	2,710,750	2,800,700	4,200,320
Active ingredient - Consortium of *Bacillus* species						
*B. subtilis + P. megaterium*	6,909		252,850	20,090		39,720
*B. thuringiensis + Brevibacillus laterosporus*					15,950	344,810
*B. subtilis + B. licheniformis*	847,370	917,400	1.162,760	1.161,100	975,050	792,780
*B. subtilis + B. velezensis*			67,110	51,670	58,730	243,390
*Bacillus paralicheniformis + B. subtilis*					27,290	90,410
*B. amyloliquefaciens + B. velezensis*						69,990
*B. amyloliquefaciens + B. subtilis*			334,870	165,140	219,640	57,350
*B. pumilus + B. subtilis + B. velezensis*			69,370	519,300	1,073,650	3,036,750
*B. amyloliquefaciens + B. pumilus + B. subtilis*	57,030	204,950	399,700	658,12	580,810	35,900
*B. amyloliquefaciens + B. subtilis + B. thuringiensis*					36,850	108,170
*B. amyloliquefaciens + B. thuringiensis + B. velezensis*				710	246,540	748,540
*B. amyloliquefaciens + B. pumilus + B. subtilis + P. megaterium*			6,420	7,900	30	0,190
Consortium of *Bacillus* and fungi						
*B. amyloliquefaciens + Trichoderma* sp.	154,930	503,170	720,390	838,340	798,330	1,353,730
*B. velezensis + Trichoderma* spp.					50,020	177,560
*B. subtilis + Trichoderma* sp.						43,600
*B. subtilis + B. licheniformis + Paecilomyces lilacinus*	96,250	420,200	551,560	397,910	446,670	771,440
*B. amyloliquefaciens + B. subtilis + Trichoderma asperellum + Trichoderma harzianum*					57,670	36,560
*B. subtilis + Trichoderma* sp. *+ P. lilacinus + Arthrobotrys oligospora + Pochonia chlamydosporia*	1,670	28,650	198,020	124,300		128,110
Potential treated area total (hectare) with *Bacillus-*based products	7,576,610	9,591,760	14,101,930	18,097,660	25,991,710	37,194,240
Total of PAT (hectare) with biological control-based products	22,186,000	26,632,000	42,549,000	52,056,000	65,724,000	86,937,000

**Fig. 3:**
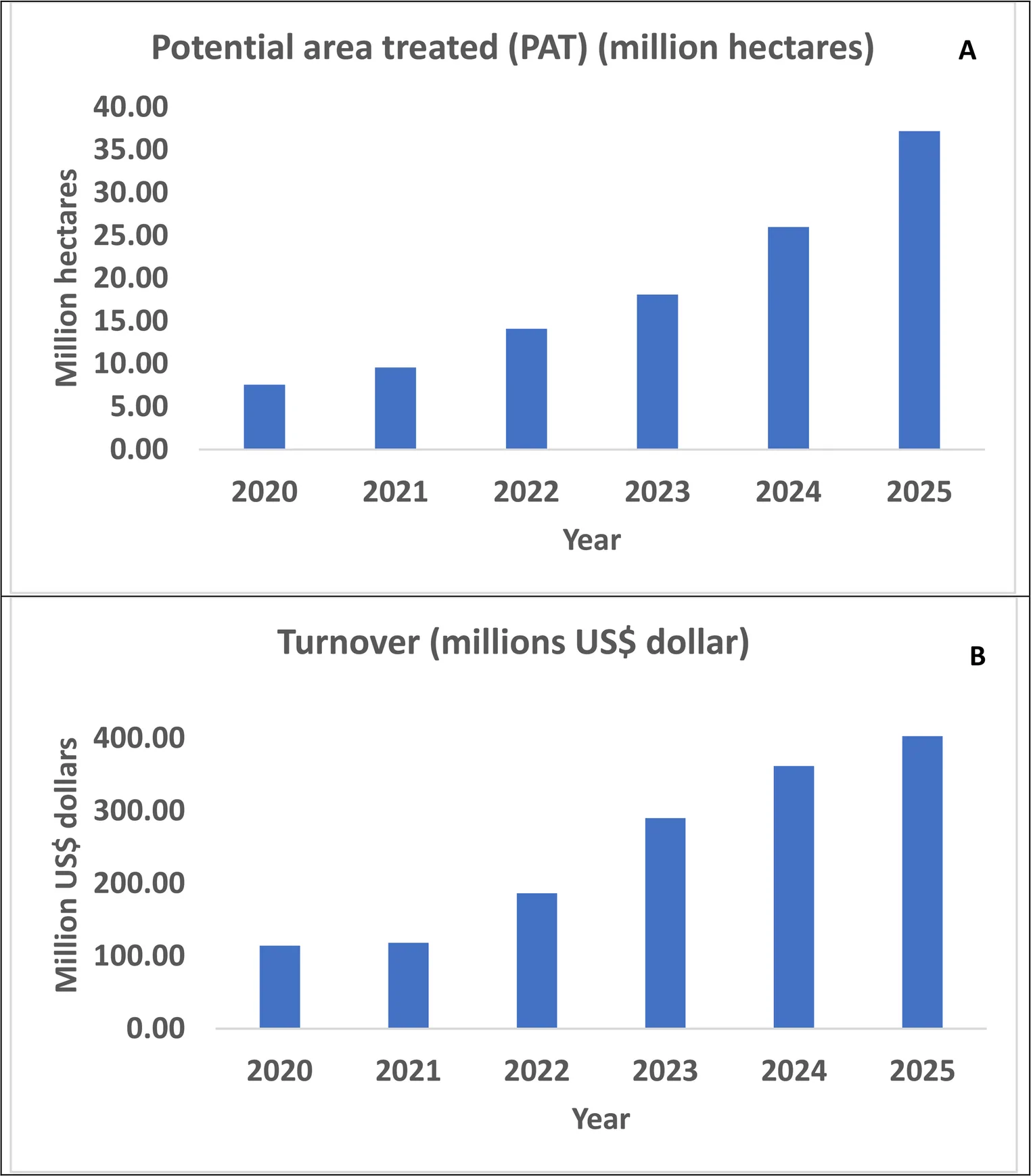
Growth trends in the potential area treated (**A**) and turnover (**B**) of *Bacillus*-based products for plant disease control in Brazil between 2020 and 2025

The evolution of the potential area treated (PAT) with *Bacillus*-based products for plant disease control in Brazil between 2020 and 2025 was remarkable (Tables 3 and 4; Fig. 3A). The PAT surged dramatically from 7,576,610 hectares in 2020 to 37,194,240 hectares in 2025, representing a staggering increase of approximately 391% over this period.

The most commercially available species for plant disease control was *B. amyloliquefaciens*, with a PAT of 13,871,490 hectares in 2025, followed by *B. subtilis*,* B. pumilus*,* C. firmus*,* B. velezensis*, and *B. methylotrophicus*, with PAT of 3,352,440; 2,861,570; 1,624,020; 1,598,270; and 1,591,680 hectares, respectively. The PAT for *Bacillus thuringiensis*, used primarily for pest control but also as bionematicides, also increased during this period, reaching 4,200,320 hectares in 2025.

There is also a trend towards an expanding market for consortium-based products containing *Bacillus* species and *Bacillus* spp. plus antagonistic fungi between 2020 and 2025 (Table 4). Consortia made up of *Bacillus* species account for a PTA of 5,568,000 hectares in 2025. The most important consortium is that consisting of *B. pumilus* + *B. subtilis* + *B. velezensis*, covering 3,037,750 hectares in 2025. On the other hand, when considering consortium with fungi, the one consisting of *B. amyloliquefaciens* and *Trichoderma* reached 1.35 million hectares (Table 4).

When considering *Bacillus*-fungi consortium, the main fungal genera used are *Trichoderma* and *Paecilomyces*. PATs were 1,574,890 and 771,440 hectares in 2025, for the consortia systems with *Trichoderma* and *Paecilomyces*, respectively. Another important consortium with fungi is: *B. subtilis* + *Trichoderma* sp. + *P. lilacinus* + *Arthrobotrys oligospora* + *Pochonia chlamydosporia*, with a PAT of 128 thousand hectares (Table 4).

### Growth trends in the turnover of *Bacillus*-based products for plant disease control in Brazil between 2020 and 2025

The turnover with crop protection (chemical, and biological control products) was US$13,775, US$12,510, US$15,711, US$21,364, US$19,915, and US$18,395 million dollars, for the years 2020, 2021, 2022, 2023, 2024, and 2025, respectively, corresponding to an 8% compound annual growth rate (CAGR) in the turnover between 2019 and 2025. However, between the 2024 and 2025 crop seasons, there was a 7.6% reduction in the total market value of crop protection (Table 3).

The turnover rate for products containing biological control agents for crop protection in Brazil was US$271.63, US$291.89, US$469.26, US$697.32, US$784.41, and US$855.68 million dollar, while for *Bacillus*-based products it was US$114.31, US$118.30, US$186.72, US$290.12, US$362.02, and US$403.17 million dollar, for the years 2020, 2021, 2022, 2023, 2024, and 2025, respectively. Thus, the turnover percentages for *Bacillus*-based products in relation to total sales of biological control-based products were 42%, 41%, 40%, 42%, 46%, and 47%, for the years 2020, 2021, 2022, 2023, 2024, and 2025, respectively (Tables 3 and 5).

**Table 5:** Turnover of *Bacillus*-based products and with total of biological control agents based-products in Brazilian agriculture from 2020 to 2025 (millions of US dollars)

Active ingredient - One species of Bacillus	2020	2021	2022	2023	2024	2025
*Bacillus amyloliquefaciens*	13.53	19.98	33.52	71.76	156.16	172.01
*Bacillus subtilis*	35.33	25.65	41.51	67.44	59.55	36.27
*Bacillus pumilus*	3.45	1.15	4.59	9.31	11.74	25.80
*Bacillus firmus (Cytobacillus firmus)*	0.30	3.04	3.32	10.21	9.33	5.91
*Bacillus velezensis*	0.02	1.33	2.88	5.46	8.94	15.85
*Bacillus methylotrophicus*	8.02	11.38	18.46	21.84	15.97	13.49
*Priestia megaterium*						0.01
*Bacillus licheniformis*						0.06
*Bacillus* sp.	3.64	3.25	1.05	1.10	0.01	
*Bacillus thuringiensis*	18.29	13.53	17.74	29.29	27.81	37.43
Active ingredient - Consortium of *Bacillus* species						
*B. subtilis + P. megaterium*	0.13		3.92	0.62		0.46
*B. thuringiensis + Brevibacillus laterosporus*					0.23	2.25
*B. subtilis + B. licheniformis*	26.52	27.88	36.14	45.45	33.78	20.76
*B. subtilis + B. velezensis*			0.91	1.15	1.14	3.86
*Bacillus paralicheniformis + B. subtilis*					0.40	1.52
*B. amyloliquefaciens + B. velezensis*						0.85
*B. amyloliquefaciens + B. subtilis*			1.28	0.62	1.29	0.26
*B. pumilus + B. subtilis + B. velezensis*			0.41	4.99	8.90	25.94
*B. amyloliquefaciens + B. pumilus + B. subtilis*	1.50	1.60	2.65	5.39	4.80	0.25
*B. amyloliquefaciens + B. subtilis + B. thuringiensis*					0.64	1.14
*B. amyloliquefaciens + B. thuringiensis + B. velezensis*				0.01	3.87	11.49
*B. amyloliquefaciens + B. pumilus + B. subtilis + P. megaterium*			0.07	0.08	0.00	0.00
Consortium of *Bacillus* and fungi						
*B. amyloliquefaciens + Trichoderma* sp.	1.09	2.23	4.10	6.10	8.32	9.50
*B. velezensis + Trichoderma* spp.					0.16	0.83
*B. subtilis + Trichoderma* sp.						0.55
*B. subtilis + B. licheniformis + Paecilomyces lilacinus*	2.48	7.08	12.71	8.29	8.87	15.02
*B. amyloliquefaciens + B. subtilis + Trichoderma asperellum + Trichoderma harzianum*					0.09	0.05
*B. subtilis + Trichoderma* sp. *+ P. lilacinus + Arthrobotrys oligospora + Pochonia chlamydosporia*	0.01	0.20	1.46	1.02		1.60
Turnover total (x millions of US dollar) with *Bacillus*-based products	114.31	118.30	186.72	290.12	362.02	403.17
Total of turnover (x millions of US dollar) with biological control agents-based products	271.63	291.89	469.26	697.32	784.41	855.68

These figures indicate a 21%, 23%, and 8% CAGR increases in the turnover between 2019 and 2025, for the products containing biocontrol agents, for the *Bacillus-*based products, and for the total market value of crop protection.

The turnover for *Bacillus*-based products for plant disease control in Brazil between 2020 and 2025 increased 252%. The turnover was US$ 114.31 million in 2020 and rose to US$ 403.17 million in 2025 (Tables 3 and 5; Fig. 3B).

The main commercially available species is *B. amyloliquefaciens* with a turnover of US$ 172.01 million in 2025, followed by *B. subtilis*,* B. pumilus*,* B. velezensis*, and *B. methylotrophicus* for plant disease control, with US$ 36.27, 25.80, 15.85, and 13.49 million dollars, respectively. The turnover for *Bacillus thuringiensis* also increased between 2020 and 2025, reaching US$ 37.43 million in 2025 (Table 5).

There is also a trend towards increasing turnover for consortium-based products containing *Bacillus* species and *Bacillus* spp. plus antagonistic fungi (Table 5). Consortia made up of *Bacillus* species account US$ 68.78 million in 2025. The most important consortium is that consisting of *B. subtilis*,* B. pumilus*,* B. velezensis* with US$ 25.94 million in 2025. It is important to note that in 2023 the main consortium was the one formed by *B. subtilis* + *B. licheniformis*, with US$ 45.45 million dollars. When considering consortium with fungi, the one consisting of *B. subtilis* + *B. licheniformis* + *P. lilacinus* reached US$15.02 million in 2025 (Table 5).

## Discussion

In harvest season 2024/2025, the turnover for biological products in Brazil was US$855,680,000, and in the same period, the turnover for products containing *Bacillus* in their formulation was US$403,170,000. Thus, approximately 47% of the turnover of the Brazilian market for biological products consisted of *Bacillus*-based products. This number is extraordinary for a single genus (Table 5; Fig. 3B). Over the past six years, the turnover for *Bacillus*-based products has hovered around 42% of total sales in the Brazilian market for biocontrol agents. These figures clearly confirm the importance of this group of microorganisms in the current Brazilian biocontrol market.

The increased importance of *Bacillus*-based products when considering the PAT is shown in Fig. 3A; Table 4. The PAT with biological products in 2025 was 86,937,000 ha, while the PAT considering only *Bacillus*-based products was 37,194,240 ha (Table 4), which corresponds to approximately 43% of the total area treated with biological products. The percentage share of *Bacillus*-based products in the PAT was ranged from 34% to 43% in the years between 2020 and 2025, respectively.

The number of registered commercial products containing *Bacillus* in their formulation has increased annually. In addition, they are recommended for the control of numerous important diseases, such as those caused by nematodes (Root-knot nematode/*Meloidogyne incognita*,* Meloidogyne javanica*,* Meloidogyne paranaensis*,* Meloidogyne exigua*,* Meloidogyne graminicola*; Reniform nematode/*Rotylenchulus reniformis*; Cyst nematode/*Heterodera glycines;* Root-lesion nematode/*Pratylenchus brachyurus*,* Pratylenchus zeae;* Burrowing nematode /*Radopholus similis)*, fungi (Anthracnose/*Colletotrichum truncatum*, *Colletotrichum lindemuthianum*,* Colletotrichum gloeosporioides*,* Colletotrichum dematium;* Target spot*/Corynespora cassiicola;* Coffee leaf rust*/Hemileia vastatrix;* Asian soybean rust/*Phakopsora pachyrhizi*; Powdery mildew/*Sphaerotheca fuliginea*,* Podosphaera fuliginea*,* Erysiphe polygoni*,* Uncinula necator*, *Sphaerotheca macularis*,* Leveillula taurica*,* Blumeria graminis*,* Sphaerotheca pannosa;* White mold/*Sclerotinia sclerotiorum;* Damping-off, root rot, Rhizoctonia disease/*Rhizoctonia solani*; Charcoal rot/*Macrophomina phaseolina*; Gray mold/*Botrytis cinereal;* Black sigatoka/*Mycosphaerella fijiensis;* Black rot/*Ceratocystis paradoxa;* Areolate mildew/*Ramularia areola*; Brown spot /*Cercospora coffeicola;* Damping-off, root rot, yellowing/*Pythium ultimum;* Fusarium wilt/*Fusarium oxysporum;* Dry root rot, fusariosis/*Fusarium solani;* Stalk rot*/Fusarium moniliforme;* Rice blast/ *Magnaporthe oryzae;* Stalk rot/*Fusarium verticillioides;* Early blight/*Alternaria solani;* Phoma leaf spot/*Phoma costaricensis;* Brown spot /*Cercospora coffeicola;* Purple blotch*/Alternaria porri;* Cercospora leaf blight/*Cercospora kikuchii;* Rust diseases/ *Puccinia polysora*,* Physopella zeae*,* Puccinia sorghi*,* Puccinia kuehnii*,* Puccinia melanocephala*,* Puccinia hordei*,* Puccinia triticina;* Cercospora leaf blight/*Cercospora kikuchii;* Tan spot/ *Drechslera tritici-repentis*; Septoria brown spot/*Septoria glycines;* Stalk rot/*Stenocarpella maydis;* Postbloom fruit drop /*Colletotrichum acutatum;* Late blight/*Phytophthora infestans;* Downy mildew/*Pseudoperonospora cubensis;* White spot/*Phaeosphaeria maydis*; Alternaria leaf blight/*Alternaria dauci);* bacteria (Common scab/*Streptomyces scabiei;* Citrus canker/*Xanthomonas citri* subsp. *citri;* Black rot/*Xanthomonas campestris;* Bacterial halo blight*/Pseudomonas syringae* pv. *garcae*; Bacterial wilt/*Ralstonia solanacearum;* Bacterial Leaf streak/*Xanthomonas vasicola* pv. *vasculorum)*, and several other diseases (Table 1). *Bacillus*-based products are applied to a wide large of crops through seed treatment, drench, planting furrow, and foliar application.

The importance of *Bacillus*-based products in the Brazilian market is attributable to the biological and functional traits of this genus, as many *Bacillus* strains exhibit nematocidal, insecticidal, and fungicidal action, in addition to promoting plant growth and alleviating abiotic stresses. Moreover, *Bacillus* acts through highly diverse and complementary mechanisms of action. The ability to produce endospores enables the development of stable formulations with a shelf-life exceeding two years.

Considering the potential of *Bacilli* in not only controlling plant diseases but also promoting plant growth and alleviating water, saline, and thermal stresses, in addition to exhibiting a certain compatibility with chemical pesticides [1–19, 29, 31, 33, 171], the number of *Bacillus* species to be investigated and explored is expected to increase in the coming years. Moreover, *Bacilli* are likely to play an increasing role in strategies aimed at reducing the use of nitrogen, phosphate, and potassium fertilizers; consequently, additional species and strains will be explored for this purpose.

A fundamental driver for expanding the exploration of new *Bacillus* species and strains lies in their capacity to produce metabolites that can be formulated into biochemical and, potentially, bioinspired synthetic fungicidal products. However, this strategy must be responsibly explored to avoid problems historically associated with synthetic chemical fungicides, such as the selection of resistant pathogens populations to their metabolites as well as ecotoxicological and toxicological problems.

We believe that the most effective way to fully harness *Bacillus* is not through the isolated application of their metabolites, but rather through formulations that combine these compounds with living cells, whether in vegetative form or as endospores. Under these conditions, *Bacillus* spp. can express their full biological and ecological potential, delivering durable benefits for both agriculture and the environment.

## Conclusion

The Brazilian market analysis presented here demonstrates that these biological attributes have translated into rapid and sustained commercial adoption. The exponential growth in potential area treated (≈ 391%) and turnover (≈ 252%) between 2020 and 2025 positions Brazil as one of the most dynamic global hubs for *Bacillus*-based bioproducts. The predominance of species such as *B. amyloliquefaciens*, *B. subtilis*, *B. velezensis*, and *B. pumilus* reflects both their biological efficacy and the robustness of scientific evidence supporting their use. Moreover, the increasing adoption of multi-strain and multi-kingdom consortia signals a paradigm shift toward more ecologically informed formulations that more closely emulate natural suppressive microbiomes.

Market maturation reflects a broader regulatory refinement, characterized by stricter regulatory requirements for strain-level identification, improved taxonomic resolution, and higher-quality market surveillance. Together, these changes have contributed to greater transparency, consistency, and scientific rigor in product registration, aligning Brazil with international regulatory standards and enhancing farmer confidence and long-term market stability.

Looking ahead, the trajectory of *Bacillus*-based biocontrol is expected to accelerate further. Advances in genomics, metabolomics, and systems biology will enable more precise strain selection, optimization of metabolite profiles, and rational design of microbial consortia tailored to specific crops, pathogens, and environmental contexts. In parallel, the integration of *Bacillus* spp. into digital agriculture, predictive disease modeling, and climate-resilient production systems will expand their functional relevance beyond mere disease suppression, positioning *Bacillus*-based products as key biological components of Integrated Crop Protection systems and holistic crop health management.

In the context of global challenges - including climate change, pesticide resistance, regulatory restrictions on chemical inputs, and societal demand for sustainable food production - *Bacillus*-based products represent not merely an alternative, but a central pillar of future plant protection strategies.

The Brazilian experience documented in this study offers a compelling model for other agricultural regions, demonstrating how scientific knowledge, regulatory evolution, and market dynamics can converge to scale biologically based solutions without compromising productivity. As such, *Bacillus* spp. are poised to remain at the forefront of sustainable plant disease control in the coming decades.

## Data Availability

Data are available upon request.
